# Biomimetic nanoparticles in ischemic stroke therapy

**DOI:** 10.1186/s11671-023-03824-6

**Published:** 2023-03-11

**Authors:** Zihao Liu, Qian Xia, Dengzhen Ma, Zhihai Wang, Longji Li, Min Han, Xianyong Yin, Xiaoshuai Ji, Shan Wang, Tao Xin

**Affiliations:** 1 Department of Neurosurgery, Shandong Provincial Hospital, Shandong University, Jinan, 250021 China; 2 Department of Endocrinology, Qilu Hospital, Cheeloo College of Medicine, Shandong University, Jinan, 250012 China; 3 Department of Neurosurgery, Shandong Provincial Qianfoshan Hospital, Shandong University, Jinan, 250021 China; 4 Department of Neurosurgery, The First Affiliated Hospital of Shandong First Medical University and Shandong Provincial Qianfoshan Hospital, Jinan, 250014 China; 5 Shandong Key Laboratory of Reproductive Medicine, Department of Obstetrics and Gynecology, Shandong Provincial Hospital Affiliated to Shandong First Medical University, Jinan, 250021 Shandong China; 6 Medical Science and Technology Innovation Center, Shandong First Medical University and Shandong Academy of Medical Sciences, Jinan, 250117 China

**Keywords:** Ischemic stroke, Membrane camouflage, Biomimetic nanoparticles, Drug delivery system

## Abstract

**Abstract:**

Ischemic stroke is one of the most severe neurological disorders with limited therapeutic strategies. The utilization of nanoparticle drug delivery systems is a burgeoning field and has been widely investigated. Among these, biomimetic drug delivery systems composed of biogenic membrane components and synthetic nanoparticles have been extensively highlighted in recent years. Biomimetic membrane camouflage presents an effective strategy to prolong circulation, reduce immunogenicity and enhance targeting. For one thing, biomimetic nanoparticles reserve the physical and chemical properties of intrinsic nanoparticle. For another, the biological functions of original source cells are completely inherited. Compared to conventional surface modification methods, this approach is more convenient and biocompatible. In this review, membrane-based nanoparticles derived from different donor cells were exemplified. The prospect of future biomimetic nanoparticles in ischemic stroke therapy was discussed.

**Graphic abstract:**

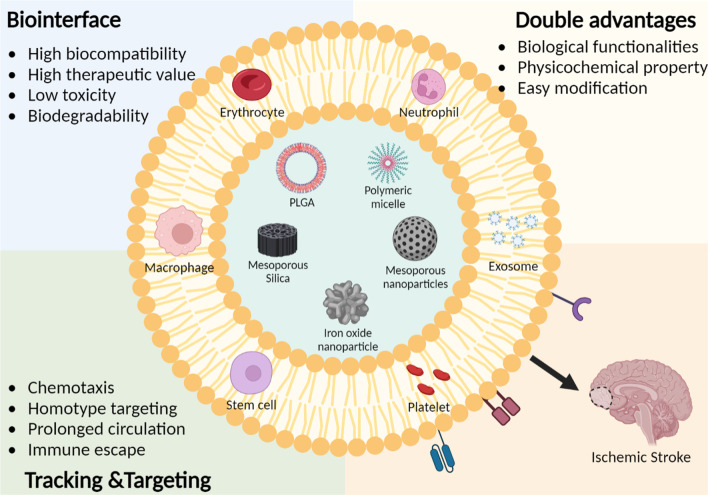

## Introduction

Ischemic stroke (IS), one of the most severe neurological disorders caused by cerebrovascular occlusion, is the leading cause of death worldwide with most of survivors being long-term disabled [1]. Over the past three decades, the prevalence and incidence rates of IS have significantly increased as global population aged [2, 3]. According to the etiologies, ischemic stroke is mainly attributed to four causes as follow: atherosclerosis, cardiogenic embolism, small vessels occlusion and undetermined pathogeny [4]. The risk factors of thrombogenesis include diseases that cause vascular endothelial damage and hemodynamics change (velocity of flow, viscosity) [5]. With respect to its epidemiological importance, there are two main strategies to reduce the global burden of IS: early prevention and effective treatment [6].

According to the latest guidelines released by American Stroke Association, the standard therapy for acute ischemic stroke is recommended as early recanalization achieved by intravenous thrombolysis or mechanical thrombectomy [7]. However, the therapeutic time windows for thrombolysis and thrombectomy are relatively narrow (within 4.5 and 6 h from onset, respectively) [8, 9] and only 2–7% patients can benefit from them [10]. For the majority who miss the optimal time window, neuroprotection and neurorestoration are indispensable for rescuing the ischemic penumbra. Unfortunately, the commonly prescribed neuroprotective drugs including Edaravone, Oxiracetam and Citicoline are unable to show satisfactory in situ bioavailability owing to the relatively low Blood Brain Barrier (BBB) permeability [11–13]. Typically, BBB can selectively impede the paracellular diffusion of more than 98% small molecules and almost all large molecules [14], which extensively restricts the clinical translation of other therapeutic reagents. Therefore, novel approaches that address the current shortage of IS management are urgently needed.

Nanomedicine, the interdisciplinary science focusing on the medical application of nanotechnology, has made remarkable advances in cerebral disorders therapy. Compared to conventional medication administration route, it is well acknowledged that nanomedicine has the advantages including reduced circulatory clearance, prolonged half-time, sustained drug release and minimized side effects [15]. Currently, a vast variety of nanoparticles have been exploited in IS therapy, including inorganic nanomaterials, organic nanomaterials, metallic nanomaterials and synthetic nanomaterials. Among all kinds of biomaterials, biomimetic membrane-based nanoparticles camouflaged with cell membrane or exosomes-derived vesicles have become the most promising drug delivery systems (DDSs) due to their excellent biocompatible and biodegradable properties [16]. Additionally, the utilization of biogenic membranes to coat nanoparticles offers advantages including long circulation, low immunogenicity and BBB penetration [17]. As mentioned before, BBB is the main obstacle for intracranial drug delivery despite the fact that BBB is transitorily disrupted during ischemia and reperfusion [18]. In order to increase the drug concentration in local tissue effectively, biomimetic membrane-based DDS coated with various BBB transport vectors (such as cell-penetrating peptides, antibodies, ligand-mimicking peptides and magnetic molecules) are widely investigated. Indisputably, nanomedicine, especially the biomimetic nanomedicine, has played an increasingly important role in cerebral disorders therapy, enabling new strategies for the medical intervention of ischemic stroke [19].

In this review, the biomimetic membrane-based DDS that has been proposed for IS therapy was delved. The physiopathology of IS was elaborated to improve understandability since various biomimetic nanoparticles are designed for targeting the specific pathological processes of IS. The superiorities of biomimetic nanoparticles in drug delivery were itemized according to their donor cell classifications. Finally, their novelty was highlighted, and the current challenges and future prospect were discussed in hopes of advancing the studies of biomimetic nanomedicines for treating IS.

## Physiopathology of ischemic stroke

**Fig. 1:**
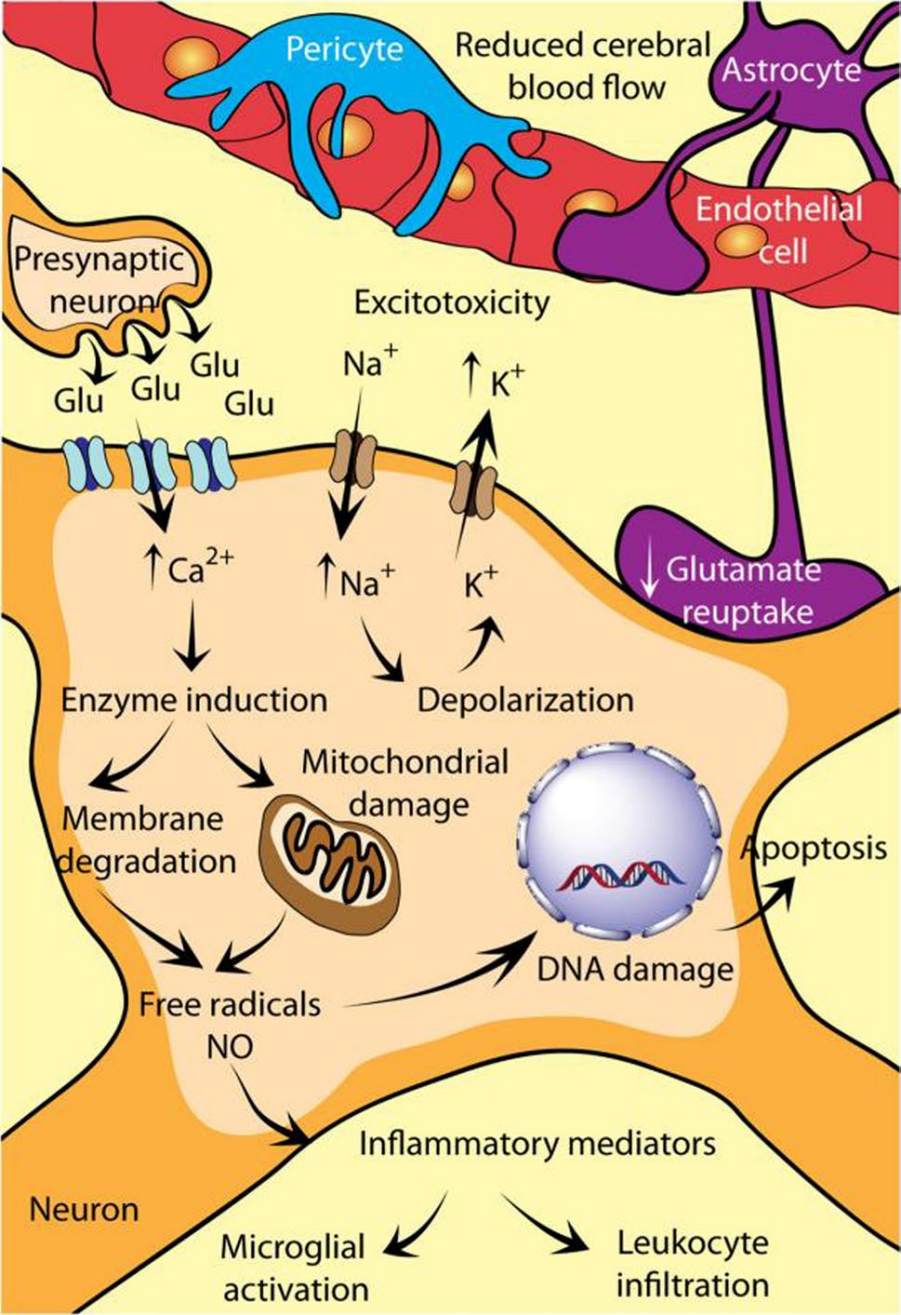
Involvement of molecular cascade during the pathogenesis of ischemic stroke. Refereed from [22] with permission obtained from Copyright Clearance Centre

Following cerebrovascular blockage, the deprivation of oxygen and energy supply in ischemic lesions activates several deleterious pathways. The disturbance of cellular homeostasis including excitotoxicity, oxidative and nitrative stress, inflammation, and inevitable neural apoptosis causes the formation of infarction and penumbra (Fig. 1). Ischemic penumbra represents the transition zone around infarction core, where reversibly injured neurons can be restored after prompt reperfusion [20]. Since the penumbra region is expanding after ischemia and self-regeneration cannot occur in cerebral tissues, salvaging as much penumbral neurons as possible has been the priority in IS rehabilitation [21].

### Ion homeostasis and glutamatergic system

Brain function depends exquisitely on oxygen for ATP generation and energy metabolism [23]. The weight of brain accounts for only ~ 2% of body, while the brain oxygen consumption at rest is more than 20% of body [24]. When energy failure occurs, the Na^+^/K^+^ ATPase pumps and Ca^2+^ pumps are compromised immediately, resulting in cell membrane depolarization along with dramatic Na^+^/Ca^2+^ influx and K^+^ outflux [25]. The disrupted ion homeostasis attributable to Na^+^, K^+^, Ca^2+^ and Cl^−^ translocation leads to consequent water influx and cytotoxic cell swelling [26, 27]. Calcium has been well recognized as a key participant in mitochondrial apoptotic pathway. The initial calcium influx can induce secondary calcium overload in cytoplasm via glutamate release [28]. The intracellular Ca^2+^ accumulation further activates calcium-dependent enzymes including proteases, lipases and nucleases, which eventually leads to the initiation of caspase-dependent cell death [29].

Glutamate is the most predominant excitatory neurotransmitter in central nervous system and specifically activates ionotropic glutamate receptors such as NMDA (*N*-methyl d-aspartate) and AMPA (α-amino-3-hydroxy-5-methyl-4-isoxazolepropionic acid) receptors [30]. Glutamate receptor is composed of subunits, which exhibited differential expression pattern in different anatomical regions and cell types [31]. Normally, the activation of ionotropic glutamate receptors is highly regulated due to the low extracellular glutamate concentration maintained by glutamate uptake system [32]. Following ischemia, the reuptake of glutamate by EAATs (excitatory amino acid transporters) expressed on astrocytes and endothelia cells is impeded [33], and the increased intracellular Ca^2+^ concentration stimulates rapid release of glutamate from presynaptic neurons [28]. Consequently, the continuous activation of ionotropic glutamate receptors triggers secondary influx of Ca^2+^ and ultimately leads to excitotoxicity. Metabotropic glutamate (mGlu) receptors, which belong to GPCR (G-protein coupled receptor) family, also play indispensable roles in the glutamatergic system after IS. There are eight subtypes of mGlu receptors divided into three groups (group I, II and III), and all of them have been postulated as potential targets for excitotoxicity prevention [34–36]. As negative regulators of glutamate, mGlu receptors can be activated by various agonists and exert inhibitory effect indirectly on glutamatergic transmission. This mechanism is considered to be devoid of side effect compared to glutamate scavenging strategy [37]. Additionally, it is reported that mGlu receptors are involved in neuroinflammatory response and can exhibit neuroprotective properties by reducing glial reactivity and promoting neurotrophic phenotype of microglia cells [38]. Given all of that, ion homeostasis and glutamatergic system are inextricably connected with excitotoxicity. Therapeutic reagents intervening those specific pathways are responsible for the neuroprotective effects of ischemic stroke therapy, and the most commonly used drugs to alleviate excitotoxicity include calcium channel blockers (CCBs), calcium chelators, glutamate antagonists and GABA (gamma amino butyric acid) agonists.

### Oxidative and nitrative stress

Brain has relatively low antioxidant activity and is vulnerable to reactive species. As the essential reactive species generated during physiological metabolism and pathological condition, ROS (reactive oxygen species) and RNS (reactive nitrogen species) play pivotal roles in the cascade of cerebral ischemic injury [39]. Typically, ROS and RNS families is comprised of superoxide (O_2_^−^), hydrogen peroxide (H_2_O_2_), hydroxyl radical (OH^−^), singlet oxygen (^1^O_2_) and nitric oxide (NO), which are maintained in low concentration levels by intrinsic antioxidant defense mechanism [40–42]. Antioxidant enzymes including superoxide dismutase (SOD), catalase (CAT) and peroxidase (POD) are pivotal components in biological systems. They can catalyze the disproportionation of superoxide anion radicals to generate oxygen and hydrogen peroxide, and catalyze the decomposition of hydrogen peroxide into harmless water and oxygen [43]. Upon ischemia and reperfusion, the formation of free radicals was significantly increased and the equilibrium between endogenous defense and ROS/RNS production systems is disrupted [41]. Mitochondrial ROS generating enzymatic systems including NADPH oxidase (NOX), xanthine oxidase (XO) and nitric oxide synthase (NOS) are activated in the process of Ca^2+^ influx and mPTP (mitochondrial permeability transition pore) opening [24, 44]. The amplified positive-feedback signal within mitochondria further initiates secondary ROS surge and subsequently leads to oxidative stress [45]. The cellular alterations induced by oxidative stress are multifaceted. Aspects including DNA damage, lipid peroxidation, mitochondria dysfunction, ER stress and activation of apoptotic factors are implicated [46–48]. The accumulation of oxygen radicals in neurons will ultimately cause destruction of cellular macromolecules and result in the programmed cell death and neurodegeneration.

Peroxynitrite (ONOO^−^) represents as a potent cytotoxic oxidant generated from the direct non-enzymatic reaction between NO and superoxide during nitrative stress. In the process of reperfusion, formation of RNS (especially NO and peroxynitrite) in the vicinity of blood vessel is significantly increased [49]. By mediating S-glutathiolation on cysteine residues, peroxynitrite is able to activate the specific matrix metalloproteinases (MMP-2 and MMP-9) expressed in brain, which disrupts the collagen and laminin in extracellular matrix (ECM) and causes increased vascular permeability [50, 51]. Additionally, excessive RNS is recognized as a crucial part in delayed reperfusion injuries based on its cellular effects on inflammation activation, mPTP opening, mitochondrial enzymes inhibition and DNA damage [52–54]. In light of the fact that integrity of BBB vasculature is disrupted by activated MMPs, the recruitment and migration of leukocytes in ischemic penumbra are substantially enhanced, which contributes to the subsequent inflammatory cascade [55].

### Inflammation

Numerous studies have demonstrated that inflammation is a double-edged sword with both detrimental and beneficial effects during ischemic stroke. The dynamic balance between pro-inflammatory and anti-inflammatory responses has a great impact on the pathogenesis of stroke. Upon ischemia, the brain resident cells microglia and astrocytes are activated within few minutes, which produce cytokines and chemokines to trigger secondary infiltration of blood-borne inflammatory cells such as neutrophils, monocytes, T cells and macrophages [56]. The infiltration of circulating cells aggravates the release of inflammatory cytokines, chemokines and ROS, which triggers hemorrhagic transformation in sub-acute phase [57].

Microglia are the main resident immunological phagocytes accounting for nearly 10% of total brain cells [58]. There are two polarized activation modes of microglia/macrophages: M1 classic phenotype that predominantly secretes pro-inflammatory cytokines (TNFα, IL-6, IL-1β, IL-12), and M2 alternative phenotype that possesses enhanced phagocytosis capacity and promoted anti-inflammatory responses (IL4, IL10, IL13, TGFβ) [59]. In light of the fact that there are overlapping functional states of microglia, the dichotomic classification of microglia is considered to be an over-simplification. Increasing studies have demonstrated that anti-inflammatory strategies were efficient in promoting neuroprotection in acute phase. Following stroke, microglia are rapidly activated to M1 phenotype by the excitotoxic cascade and danger-associated molecular patterns (DAMPs) released from infarction region [60]. In the wake of inflammatory signal attenuation, the functionally dominant phenotypes of endogenous microglia and recruited macrophages can gradually switch to M2 phenotype in weeks [61]. The balance between two polarized phenotypes is modulated by multiple pathways and mediators, which include Pattern recognition receptor (PRR) signaling, Cytokine receptor signaling, Chemokine receptor signaling and Neurotransmitter receptor signaling [58]. Messengers including chemokine fractalkine (CX3CL1), CD200 and TREM2 (triggering receptor expressed on myeloid cells 2) participate in the cross-talk between microglia, neurons and astrocytes [62, 63].

Astrocytes represent the most abundant glial cells in brain and mainly act as mediators to maintain the homeostasis of neurons [64]. In ischemic penumbra, astrocytes are activated and manifest strong phenotypic transformation (hypertrophy, proliferation and increased GFAP (glial fibrillary acid protein) expression) from resting state to reactive state [65]. As one of the classic pathological features, reactive astrogliosis profoundly exert protective functions in ischemic stroke despite companying with inhibitory effects on recovery. Astrocytes possess abundant GSH metabolism-related enzymes and glutamate transporters (EAATs) which are essential for reducing oxidative stress and excitotoxicity [66]. As the indispensable structural part of neurovascular unit accompanying with endothelia and pericytes, astrocytes maintain the normal function of BBB. Additionally, astrocytes can secrete a variety of neurotrophic factors including brain-derived neurotrophic factor (BDNF), glial cell line-derived neurotrophic factor (GDNF), nerve growth factor (NGF), erythropoietin (EPO), vascular endothelial growth factor (VEGF), ciliary neurotrophic factor (CNTF) and neurotrophin 3 (NT-3) [67]. The production of these neurotrophic factors is enhanced after ischemia, which promotes angiogenesis, neurogenesis and axonal remodeling in penumbra region [68]. Spatiotemporal astrocyte activation states are associated with specific transcriptome programs and functions [56, 69]. During acute ischemic phase, reactive astrocytes generate pro-inflammatory mediators (such as TNF-α, IL-1, and MMPs) which leads to increased BBB permeability and edema [70, 71]. With passage of time, the astrocytes accumulated at penumbra can form structurally borders (glial scar) to restrict the entry of inflammatory cells and prevent the spread of toxic factors from infarction. Nevertheless, the axonal regeneration and functional recovery are limited [72]. Given all of that, the activation of astrocytes in ischemic stroke is a dynamic process, from hypertrophy and proliferation to glial scar formation.

## Cell membrane-based nanoparticles for IS treatment

**Fig. 2:**
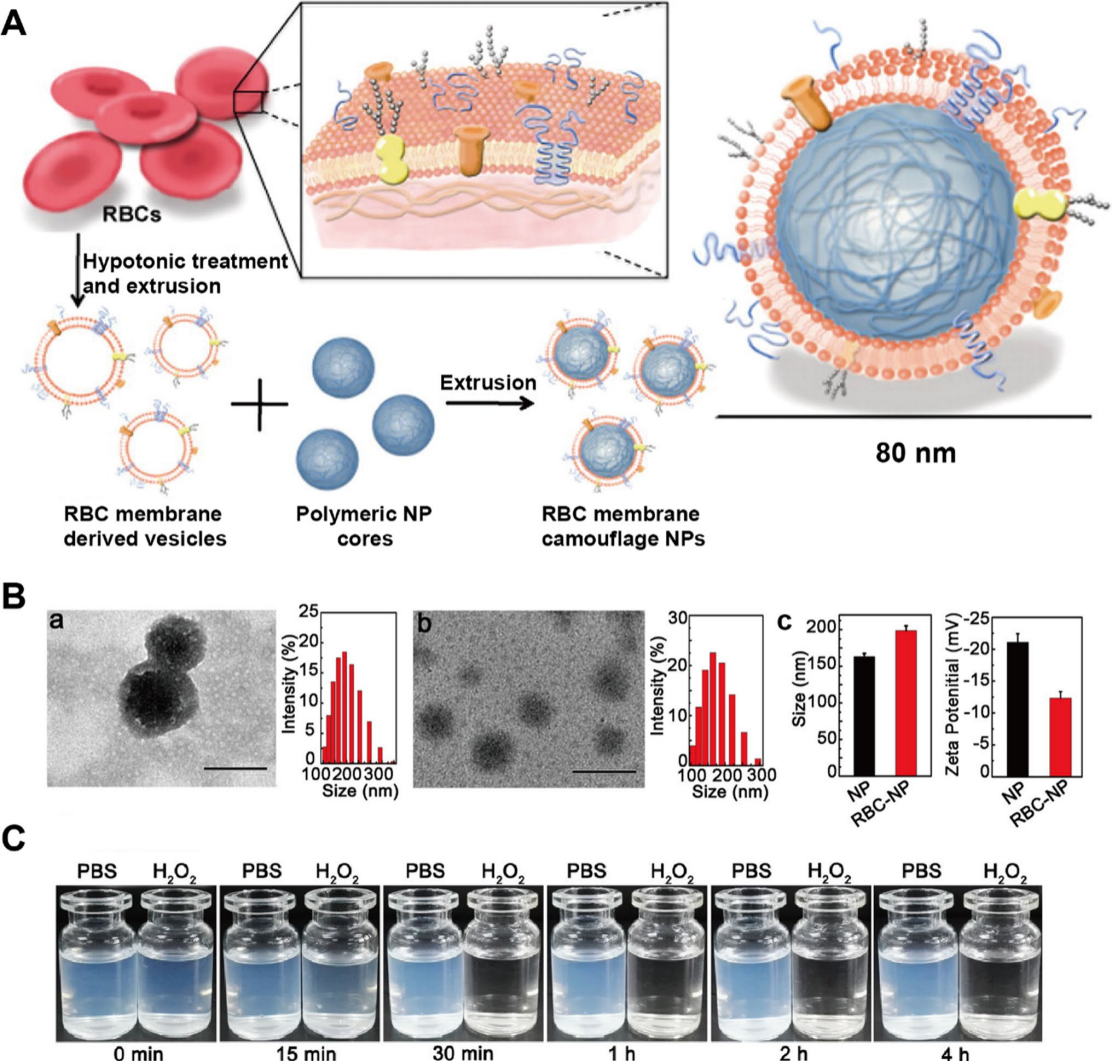
Schematic illustration of erythrocyte-based nanoparticles. **A** The fabrication procedures of RBC (red blood cell)-derived PLGA nanoparticles. **B** TEM (transmission electron microscope) images and size distribution of RBC coated nanoparticles (a) and uncoated nanoparticles (b), scale bar = 200 nm. (c) Change in the particle size and zeta potential of nanoparticles after coating with RBC membrane. **C** ROS-triggered hydrolysis of the RBC-coated nanoparticles in H_2_O_2_. Referred from [73, 79] with permission obtained from Copyright Clearance Centre

As a novel class, biomimetic DDS composed of biogenic components and synthetic nanoparticles has been extensively highlighted in recent years. The advantages of biomimetic DDS include high biocompatibility, long circulation, BBB penetration and active homing tropism. In 2011, the first biomimetic DDS camouflaged with erythrocyte membrane was developed by Zhang et al. [73] (Fig. 2A). Its elimination half-life was significantly increased for more than twice to that of the PEG-coated nanoparticles which used to be the most common strategy to improve systemic circulation time. The reason accounting for long circulation is that the biological functions of original cell membranes were inherited in the biomimetic DDS. Typically, the “self-recognition molecules” on surface of natural cell membranes or extracellular vesicles is retained, enabling nanoparticles to achieve circulatory evasion of reticulo-endothelial clearance and immune elimination [74]. Additionally, by virtue of the homing tendency of surface molecules on membrane, biomimetic DDS is endowed with unique characteristics including homologous targeting, local accumulation and prolonged retention.

As for the intrinsic nanoparticle encapsulated in biogenic membrane, its physical and chemical properties are reserved. The characteristics of original nanoparticles such as magnetism, chemical responsiveness, high drug encapsulating efficacy and sustained release capacity could be inherited in biomimetic DDS, enabling for their better therapeutic performances in IS treatment [75]. In addition, camouflage nanoparticles with biomimetic membrane also represents as a promising strategy for the surface modification of nanoparticles. Based on membrane modification technology (such as gene editing, chemical modification, membrane-liposome fusion), the biomimetic DDS could obtain opportunity to interact with specific ischemic microenvironment [76].

In the past decade, numerous cell types including erythrocyte, platelet, immune cells and stem cells were utilized in biomimetic nanoparticles. According to different donor cell sources, biomimetic DDS can be applied in diverse scenarios. The following subsections will give a detailed introduction about each type of biomimetic DDS for IS treatment.

### Erythrocyte and platelet membrane-based nanoparticles for IS treatment

Erythrocyte and platelet are the most widely utilized natural cells in biomimetic nanotechnology because of their exceptional properties such as easy availability (enormous quantity) and easy purification (lack of nuclei and organelles). The erythrocyte membrane is naturally evolved to provide tensile strength and deformability for cargo delivery (oxygen and carbon dioxide) in blood circulation [77]. Upon description of improved blood circulation time and immune evasion features of erythrocyte membrane coating, several studies highlighted its potential for drug delivery platform. Bahman et al. [78] constructed erythrocyte-derived nanoparticles conjugated with ICG (indocyanine green fluorophore) and tPA. The biomimetic DDS offered dual functionality for NIR imaging and thrombolytic capability, in which the erythrocyte membrane played an essential role in prolonged circulation time of tPA. Since erythrocyte membrane disguised nanoparticles have poor controllability of drug release and are lack of specific targeting property, Xin et al. [79] developed erythrocyte membrane coated polymer nanoparticles inserted with stroke homing peptide SHp to deliver neuroprotective agent NR2BC (Fig. 2B, C). The polymer core was modified with boronic ester which could respond to the upregulated ROS in ischemic region. As a result, the bioengineered nanoparticles prolonged systemic circulation of drug, enhanced the active homing to ischemic area and achieved controlled release. As a novel strategy to prolong drug circulation time, the utilization of cell membrane to disguise nanoparticles can completely replicate the surface antigenic diversity of source cells [80]. For instance, the immunomodulatory protein CD47 on erythrocyte surface inhibits macrophage phagocytosis and confers anti-inflammatory property through interactions with signal regulatory protein alpha (SIRPα) [81]. Other membrane proteins on the surface of erythrocytes also protect them from complement system attacks [82]. Additionally, surface modification of cell membrane is feasible and facile. Through conjugation method (chemical strategy), lipid-anchoring method (physical strategy) and transfection engineering method (genetic strategy), specific homing ligands can be inserted into erythrocyte membrane [83–85]. Therefore, it is a creditable formulation to exploit natural erythrocyte membrane as an alternative strategy for conventional PEGylation nanocoating or surface modification.

**Fig. 3:**
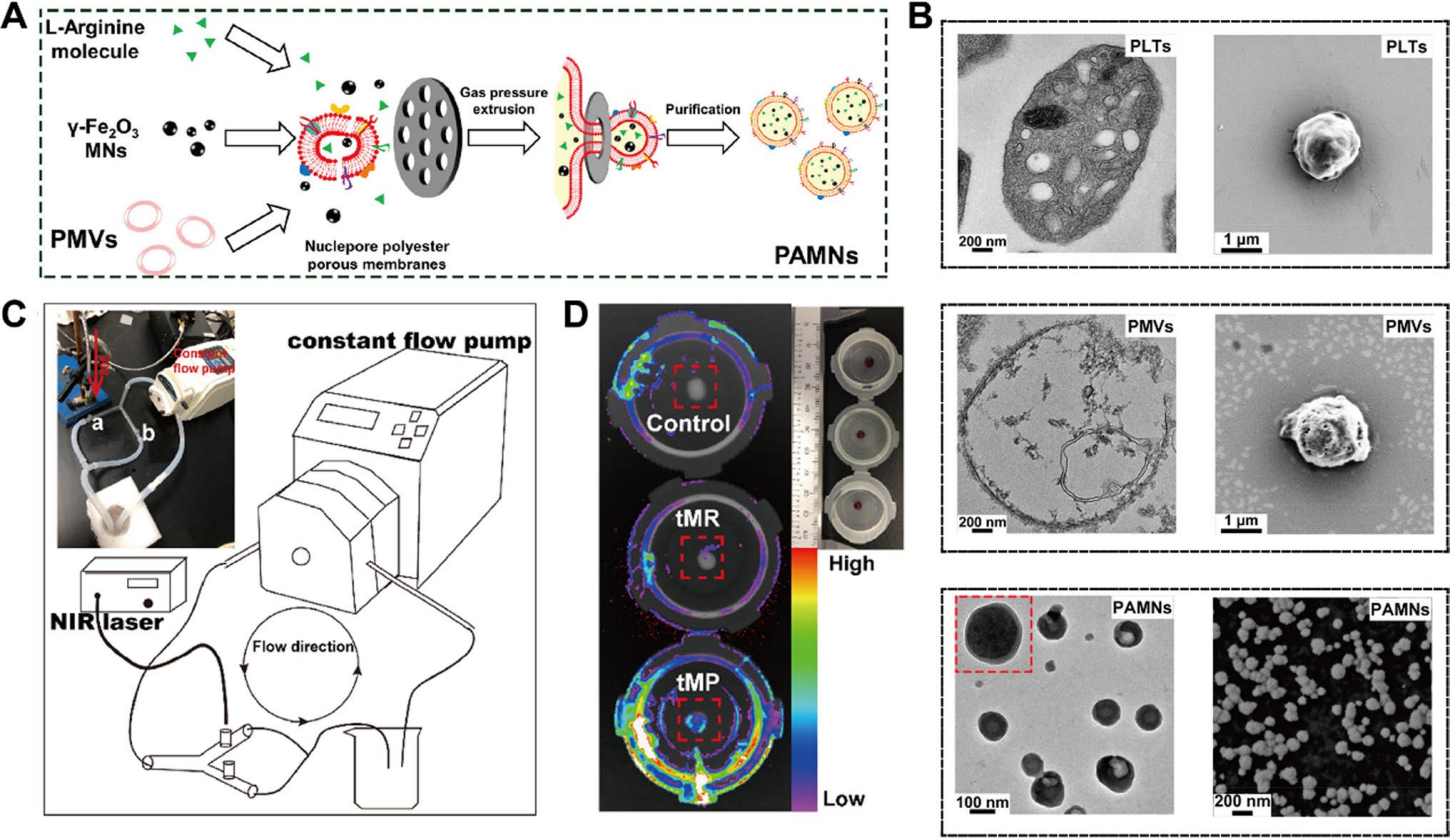
Schematic illustration of platelet-based nanoparticles. **A** The fabrication procedures (extrusion method) of platelet-derived nanoparticles. PMVs: platelet membrane envelopes; PAMNs: PMV loaded with L-arginine and γ-Fe_2_O_3_ magnetic nanoparticles. **B** TEM characterization of the internal structures of nanoparticles. **C** Thrombolysis experiment in 3D-printed vein vasculature. **D** Fluorescence imaging of labeled DiR in blood clots. tMR: (tPA/MNP@RBC membrane), tMP: (tPA/MNP@PLT membrane). Referred from [86, 87] with permission obtained from Copyright Clearance Centre

Natural platelets (PLTs) are associated with hemostasis and thrombosis. Therefore, they have a disposition to accumulate at the damaged blood vessel during thrombus formation and offer an attractive choice for fabricating biomimetic nanoparticles (Fig. 3). Based on their functions in thrombus targeting and immune escape, Gu et al. [86] constructed biomimetic nanoparticles loaded with PLTs membrane, L-arginine and γ-Fe_2_O_3_ magnetic (Fig. 3A, B). On the basis of dual navigation including thrombus anchorage and external magnetic field stimulus, the nanoparticle platform was rapidly accumulated at the ischemic injury and achieve local release of l-arginine to restore blood flow reperfusion. Likewise, Shi et al. [87] developed biomimetic nanovesicle encapsulating melanin nanoparticles (MNP) and tPA with PLTs membrane, which integrated the free radical scavenging property, photothermal conversion performance and natural thrombus-targeted capability for IS treatment (Fig. 3C, D). Compared to erythrocyte membrane-coated nanoparticles control group, the PLTs nanovesicle displayed significant adhesion and retention property to damaged blood vessels and thrombus. Apart from aggregating at damaged blood vessel, activated platelets can interact with neutrophils and aid their inflammatory infiltration into the ischemic region during IS. Zhang et al. [88] designed PLT-mimetic nanoparticles loaded with piceatannol and superparamagnetic iron oxide (SPIO) to discern, intervene and monitor activated neutrophils in acute IS. The results indicated that enhanced internalization of nanoparticles was about 3.4-fold higher than that of control. The platelet membrane–coated biomimetic drug delivery systems have been demonstrated with improved delivery performance. However, since the PLTs membrane is unable to confer BBB penetrating or ischemic penumbra targeting capacity, PLTs membrane-coated nanoparticles are not the first choice for the intracranial treatment of IS. Without exception, recent advances [89–91] in PLTs biomimetic nanoparticles were focused on the tPA adjuvant thrombolytic therapy during hyperacute phase of IS.

### Macrophage (monocyte) membrane-based nanoparticles for IS treatment

**Fig. 4:**
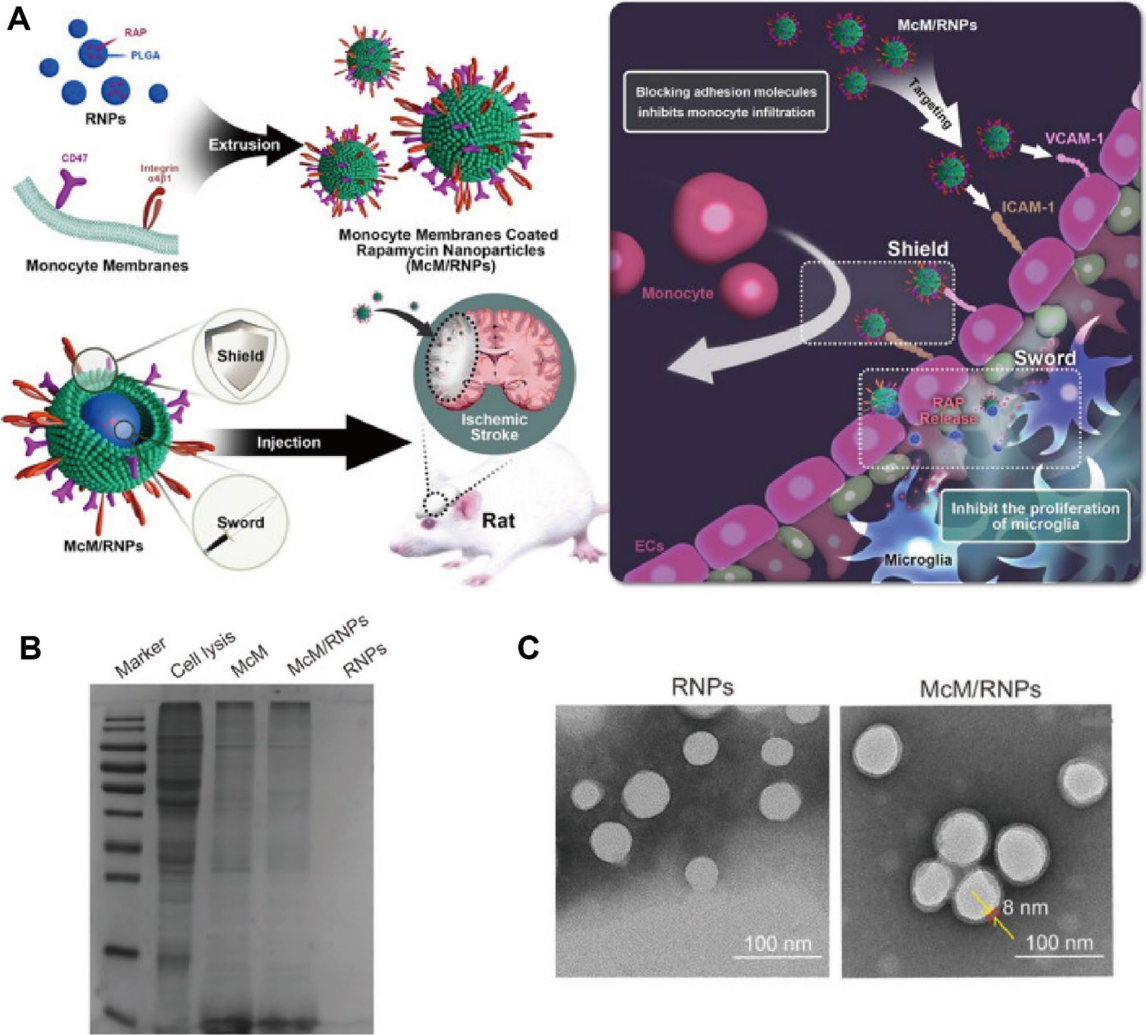
Schematic illustration of macrophage/monocyte-based nanoparticles. **A** The fabrication procedure and “shield-sword” therapeutic strategy of monocyte membrane-coated rapamycin nanoparticles (McM/RNPs). **B** The Coomassie brilliant blue stain of protein contents in whole cell lysis, monocyte membrane and McM/RNPs. **C** TEM characterization of RNPs and McM/RNPs. Refereed from [97] with permission obtained from Copyright Clearance Centre

The peripheral monocytes migrate into brain and differentiate into macrophages, which represent as an indispensable component of immune monitoring system [92]. In the process of ischemia and reperfusion, intercellular adhesion molecule-1 (ICAM-1), VCAM-1 and P-selectin on endothelial cells are overexpressed [93]. Consequently, these molecules can interact with corresponding ligands (CD11, CD18 and CD44) on leukocytes and induce leukocytes recruitment (especially for macrophages and neutrophils) from peripheral blood into the ischemic region [93]. Macrophage membrane-coated NPs have demonstrated high targeted delivery efficiency to various inflammatory diseases as well as long circulation and satisfactory therapeutic efficacy [94]. By virtue of the protein-mediated recognition, Jiang et al. [95] developed macrophage-disguised manganese dioxide (MnO_2_) nanosphere loaded with fingolimod. The half-life of encapsulated fingolimod was significantly prolonged by two times, and the biomimetic nanoparticles can accumulate actively in damaged brain. Consequently, the proinflammatory microenvironment including oxidative stress and M1 microglia transition was reversed and the ischemic penumbra was salvaged. Compared with other DDS, inorganic-based nanoparticles have the advantages of easy preparation and high loading content, but their biocompatibility is relatively poor. The results showed that coating of macrophage membrane could significantly reduce the hemolysis caused by MnO_2_ nanosphere. In addition to simply improving delivery efficiency and biocompatibility, cell membrane coating can act as a scavenger for proinflammatory factors [96]. In an example work, Wang et al. [97] developed a “nano-soldier” PLGA nanoparticles functionalized with monocyte membrane (Fig. 4A–C). The nanoconstructs were able to target and bind to endothelial cells acting as a “shield”, which competed with inflammatory leukocytes in circulation and blocked their infiltration. Subsequently, the accumulation of biomimetic nanoparticles in ischemic region achieved rapamycin release and relieved the reperfusion-induced injury. The shortcomings of polymer-based nanoparticles includes easy clearance, low specificity and low delivery efficiency. The introduction of monocyte membrane effectively improved acting homing tendency of PLGA nanoparticles and enhanced their therapeutic effects.

In addition to the navigation function, cell membrane-based nanoparticles have the advantage of facile synthesis modification such as forming hybrid biomimetic vesicle with synthetic liposome. As an exogenous vesicle, liposome is lack of targeting property and can be easily ingested by reticulo-endothelial systems in circulation. Along this line, Li et al. [98] formulated a biomimetic vesicle constituted by liposome and macrophage membrane to deliver therapeutic reagent lncRNA-EPS. The macrophage membrane fusing strategy have a great BBB penetrating and inflammatory targeting effect. In vivo data demonstrated that the engineered vesicles could specifically target to inflammatory sites and increased lncRNA-EPS levels in microglia by 77.9%, which decreased the cytotoxic capability induced by inflammatory cells and accelerated neuron regeneration.

Benefiting from the inflammatory chemotaxis capacity of macrophage membranes and their ability to infiltrate into deep tissue, macrophage/monocyte-based nanoparticles were mainly designed to abate the inflammation in IS. For instance, Chen et al. [99] utilized modern Chinese medicine and constructed macrophage membrane-based ROS-reactive nanoparticles to target inflammatory microenvironment. Altogether, these biomimetic nanoparticles encapsulated with anti-inflammatory reagents exert therapeutic effects attributing to two strategies: reducing the inflammatory infiltration from periphery and inhibiting the immunological cascade in ischemic focal site.

### Neutrophil membrane-based nanoparticles for IS treatment

**Fig. 5:**
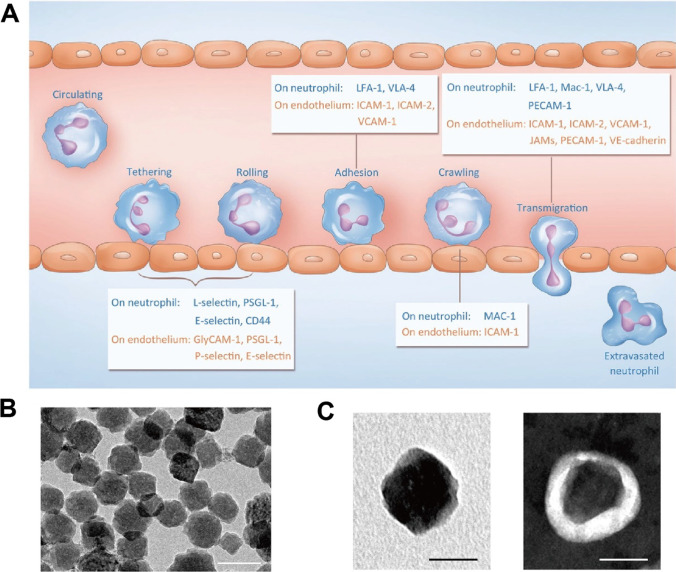
Schematic illustration of leukocyte-based nanoparticles. **A** The mechanism of nanoparticle inflammatory transmigration mediated by CAMs (Cell adhesion molecule) on leukocyte membrane. **B** TEM characterization of Prussian blue nanozyme, scale bar = 100 nm. **C** TEM images of nanozyme (left) and neutrophil membrane-coated nanozyme (right) after being stained by uranyl acetate, scale bar = 50 nm. Refereed from [76, 103] with permission obtained from Copyright Clearance Centre

Neutrophils are the most abundant leukocytes which play an essential role in acute inflammation of ischemic pathophysiological changes [100]. Like other leukocytes such as macrophages and monocytes, transmigration of neutrophils from blood to injured region is a natural process with great tropism which can be employed to deliver therapeutic reagents actively (Fig. 5A). During ischemia, increased inflammatory cytokines (such as IL-1β and TNF-α) and chemokines (including CXCL2 and CXCL12) lead to the recruitment and activation of neutrophils [101]. Neutrophils are the first cell type to arrive, and evidence have indicated that neutrophils accumulate in the ischemic penumbra cortex during all stages of IS [102, 103]. In an example work, Wang et al. [104] developed neutrophil membrane-derived nanovesicles based on the natural adhesion of neutrophils to endothelial cells. It was determined that the nanovesicle specifically targeted inflamed brain endothelium through ICAM-1 and integrin β_2_ interaction. Neuroprotective Resolvin D2 (RvD2) that enhanced the resolution of inflammation was encapsulated in. The results demonstrated that effective delivery of RvD2 to ischemic area protected brain from perfusion injury. Likewise, Zhang et al. [76] constructed mesoporous Prussian blue nanozyme coated with neutrophil membrane (Fig. 5B, C). Compared with control group, the coating of neutrophil membrane facilitated the sustained accumulation of nanoparticles in the damaged brain after stroke. During IS, accumulated detrimental factors (such as ROS and inflammatory cytokines) in infarct core erode ischemic penumbra constantly. Therefore, Chen et al. [101] proposed “nanobuffer” strategy to remodel the hostile microenvironment of ischemic core. Neutrophil-biomimetic PLGA nanoparticles were loaded with α-lipoic acid (LA) and cannabidiol (CBD) that could eliminate ROS, suppress inflammation and alleviate oxidative stress. As a result, the administration of nanobuffer neutralized the deleterious factors within infarction microenvironment and simultaneously exert neuroprotective effects to salvage penumbra.

Attributed to the receptor-mediated tropism and integrin-mediated adhesion, neutrophil membrane confers nanoparticles with homotypic targeting capacity and stability in blood circulation. Intriguingly, superficial modification mechanisms based on artificial neutrophil-related ligands were utilized in recent advances [105, 106]. The neutrophil-homing ligands (such as Mac-1 and LFA-1) drove the interaction with inflamed microvascular endothelial cells and lured DDS to damaged brain. Thus, it provides an applicative perspective for targeting therapy in IS.

### Stem cell membrane-based nanoparticles for IS treatment

Stem cells are a kind of pluripotent cells that possess self-renewal ability, multilineage differentiation ability and low immunogenicity. Neural stem cells (NSCs) have been explored for the treatments of IS because of their significant tropism to ischemic brain [107]. Combined with these benefits, optimized stem cell membrane-based biomimetic DDS allows maximum delivery efficacy. Among all the chemokines that drive NSCs tropism, CXCR4 is the most primary factor interacting with ligand SDF-1 in ischemic microenvironment. Enlighted by this chemotactic mechanism, Zhou et al. [108] developed NSCs membrane-cloaking PLGA nanoparticles. In particular, the NSCs membranes were genetically modified with overexpressed CXCR4 through lentiviral transfection. Attributed to CXCR4-engineered NSCs membrane, this approach significantly improved the drug delivery efficacy of anti-edema agent glyburide. The CXCR4-mediated chemotactic distribution was also utilized by Zhang et al. [109] to formulate nanoparticles with preferential targeting to ischemic cerebrum. Engineered mesenchymal stem cells (MSCs) membranes with overexpressed CXCR4 were applied as outer shell to neutralize the CXCL12 in overactivated brain immune microenvironment. cGAS inhibitor A151 was encapsulated in the core of polydopamine nanosphere, which displayed ROS scavenging and anti-inflammatory effects.

Stem cells including NSCs, MSCs, embryonic stem cells (ESCs) and induced pluripotent cells (iPSCs) have been acknowledged as a neurodegenerative approach to IS therapy [110]. Owing to the capacity of self-renewal, homing and multilineage differentiation, stem cells and stem cell-derived extracellular vesicles transplantation show dramatic tissue repair and regeneration potentials. Currently, almost all investigations on stem cell-based biomimetic nanoparticles were focused on cell membrane only. Among them, the moiety that play the major role are internal nanoparticles loaded with therapeutic reagent rather than the stem cells. Hence, developing novel biomimetic nanoparticles with intracellular contents retained through “Top-down” strategy may effectively improve the therapeutic capability of overall stem cell-based biomimetic nanoparticle in prospect.

## Extracellular vesicles-based nanoparticles for IS treatment

Exosomes are the extracellular vesicles derived from cells and are characterized by lipid biolayer membrane. They are 40–100 nm particles functioning as signalosomes that transmit bioactive molecules to adjacent cells for intercellular communication [111]. The most abundant bioactive substance in exosome is miRNA, which plays a pivotal role in regulating the expression level of target genes [112]. Numerous studies have shown that miRNAs in exosome can effectively promote recovery of neurological functions in stroke [113–115]. Up to now, clinical trials based on extracellular vesicles have been launched (NCT03384433, NCT05326724). As the representative, exosomes have a promising prospect in the application of biomimetic DDS.

In recent years, natural exosomes have been emerging as competent nanovehicles owing to their stability, high biocompatibility and low immunogenicity in circulation [116]. Research demonstrated that the MSC derived exosome could selectively target brain pathologies including ischemic stroke [117]. Exosome possesses abundance of adhesive proteins that readily interact with cellular membrane [118]. Given that the surface composition of exosome plays an indispensable role in clearance escape and homing capacities, incorporation of exosome membrane with nanoparticles is crucial to the application in biomimetic drug delivery. Additionally, exosomes are characterized with small size, slightly negative zeta potential and deformable cytoskeleton, which endow them the capacity to infiltrate into deep tissues [119].

**Fig. 6:**
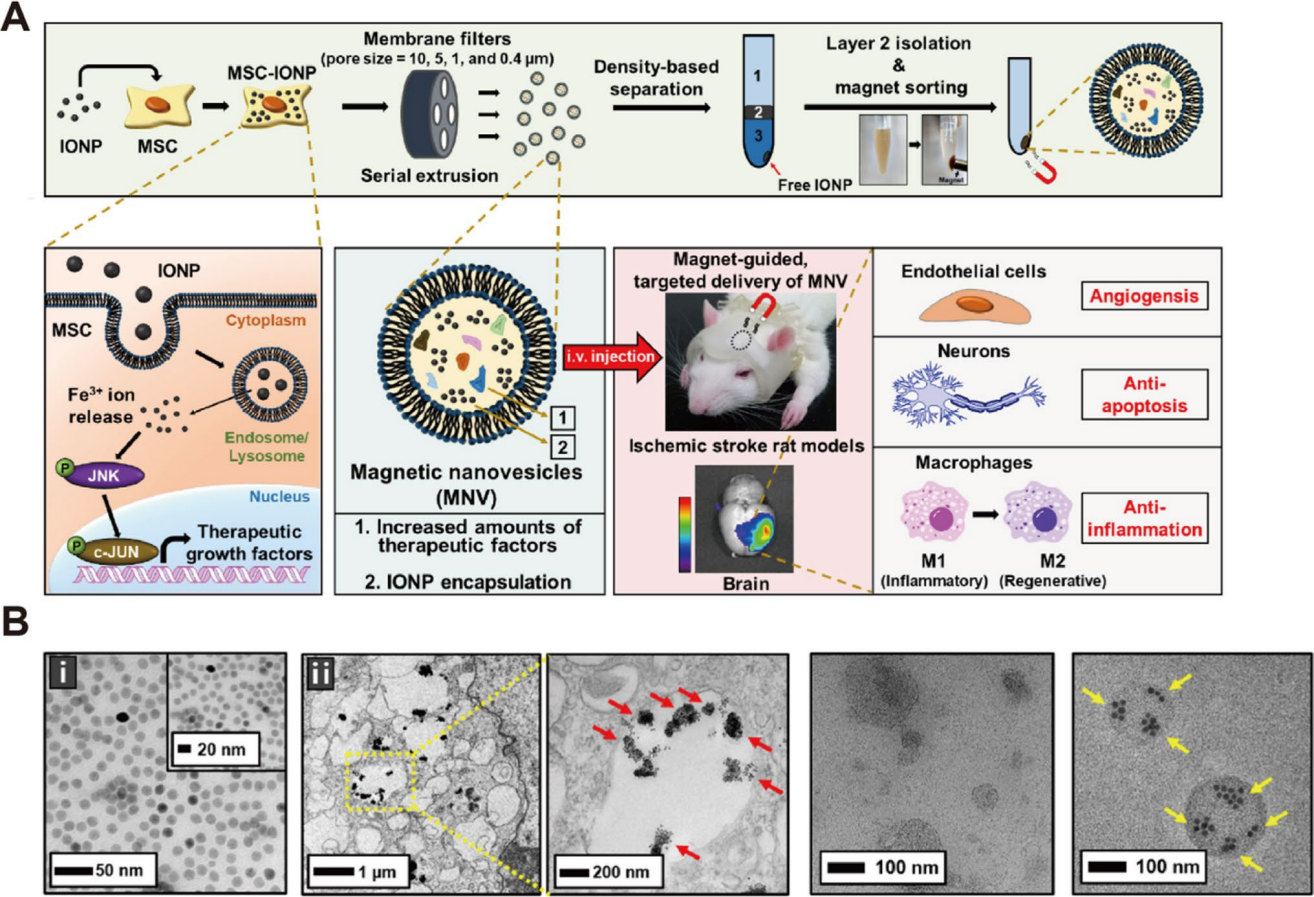
Schematic illustration of extracellular vesicle-based nanoparticles. **A** The fabrication procedure and therapeutic strategy of magnetic biomimetic nanoparticle. **B** TEM characterization of the structures of (i) IONP and (ii) MSC-IONP. **C** TEM characterization of the structures of (i) extracellular nanovesicles and (ii) magnetic extracellular nanovesicles. Referred from [122] with permission obtained from Copyright Clearance Centre

Exosome membranes have higher levels of cholesterol and sphingolipid than the corresponding donor cell membranes, making them more resistant to external interference [120]. As compared to cell membrane-based therapy, exosomal DDS may exhibit superior advantages since nanometer-sized extracellular vesicles can avoid entrapment in lung following systemic circulation [121]. Furthermore, exosome contains diverse biomolecules with therapeutic effects such as proteins, lipids and nucleic acids, which retain some of the characteristics of parent cells. Intriguingly, Kim et al. [122] developed magnetic nanovesicles derived from iron oxide nanoparticles (IONP)-harboring MSC (Fig. 6A). MSCs contained great amounts of therapeutic factors, and IONP could further stimulate the expression of angiogenic factors and growth factors. Based on the targeted delivery to ischemic region driven by magnetitic guide and MSC exosome-mimetic membrane, nanovesicles exhibited satisfactory efficacies on angiogenesis, anti-apoptosis, and anti-inflammation. Arg-Gly-Asp (RGD) peptides are the most well-known ligands that interact with integrin α_v_β_3_. Leveraging this condition, Gao et al. [123] utilized chemical method to generate RGD-C1C2-decorated extracellular vesicles (EV) that were originated from neural progenitor cells. The engineered EV nanoparticles showed enhanced targeting efficiency in infarct hemisphere compared to natural EV, and the platform exerted anti-inflammatory effects attributing to the therapeutic miRNAs within it.

At present, investigations focusing on the exosome membrane-based nanoparticles are in few numbers. In order to generate clinical doses of exosomes, extremely large-scale of cell cultures and laborious consecutive purification are required. It is considered that the small quantities of exosomes released from donor cells may limit further clinical application since conventional preparation method to collect sufficient exosome membranes for research is impractical [122]. Surprisingly, the exosome-mimetic/hybrid nanovesicles prepared by extrusion method could exhibit increased production yield for 250-fold compared to that of naturally secreted exosomes [124]. Novel technique such as 3D-culture could simulate the physiological conditions of donor cell niches and thereby significantly enhanced the exosome production efficiency [125]. In future, more studies regarding the preparation and storage of exosomes are imperative to bring these biomimetic nanoparticles a step further to clinical reality.

## Discussion

Synthetic drug delivery systems are extensively utilized to intervene the pathological mechanisms of ischemic stroke such as excitotoxicity, oxidative stress, inflammation, etc. However, the exogenous characteristics of these synthetic “invaders” may easily induce immune response and systemic clearance, which result in relatively low bioavailability and therapeutic efficacy. In the past decades, biomimetic camouflage that based on cell membranes and extracellular vesicles have emerged as novel strategy to develop multifunctional and diversified nanoparticles for IS therapy. By virtue of the surface proteins retained on biomimetic membrane, cloaking nanoparticles obtain superior properties including immune escape, specific targeting, low toxicity and long circulation. They provide attractive options for overcoming the barriers in the treatment of IS. The membranes and exosomes from a vast variety of cells have been investigated for biomimetic DDS construction, including erythrocyte, platelet, macrophage, neutrophil and stem cells. Nevertheless, the application of microglia and lymphocyte based-biomimetic nanoparticles remain limited. The reason may be linked to the fact that lymphocytes have natural tendency to accumulate at peripheral lymphoid tissue, and the defects such as high immunogenicity and difficulty of production (relatively low proliferation capacity, easy to polarize).

Looking forward, biomimetic nanoparticle systems play an increasingly important role in IS therapy. It is noted that great diligence should be taken in safety issue. Rigorous surface protein examinations (especially for glycophorin, HLA system and immunogenicity associated proteins) are needed to maximize compatibility and minimize side effects. The method to develop biomimetic nanoparticles is mainly based on two strategies: i) Modify synthetic nanoparticles with cell vesicles (bottom-up strategy); ii) Encapsulate therapeutic molecules with cell vesicles (top-down strategy) [100]. However, the overriding challenge is that simple cell membrane/extracellular vesicles are unable to display effective encapsulation. Inserting liposomes into nanovesicles could resolve the issue of low drug loading. Liposomes are the synthetic spherical systems with similar structure and properties to biological membrane. They have been extensively investigated for the development of pharmaceutical formulations (also for COVID-19 vaccine recently) [126]. Although hybrid membrane-based nanoparticles (cell membrane/exosome hybrid with liposome) have been extensively utilized in other brain disorders [127], few were reported in the treatment of ischemic stroke. It is therefore crucial to develop novel hybrid membrane-based platform since encapsulation efficiency remains as a major constraint for ultimate clinical translation of biomimetic nanoparticles.

Currently, the researches on biomimetic DDS for IS treatment are still limited to conventional small molecule drugs. Novel strategies should be considered in future studies since methods such as hyperthermia, oxygen delivery and sonothrombolysis have been proposed in IS treatment [128–130]. These methods exhibited satisfactory therapeutic effects and could be optimized with biomembrane coating strategy in future. The camouflages of biogenic membrane endow DDS with properties of passive targeting and active targeting to ischemic region, which are mainly driven by inflammation chemotaxis and ligand binding [117]. Despite strong homing tendency, insufficient drug release in lesion area may occur. Accumulating evidences have revealed the therapeutic advantages of stimulus-responsive nanoplatform for a plethora of diseases including IS [79, 131]. Nevertheless, none of them were exploited in biomimetic nanoparticles. Innovations and explorations on stimulus-responsive strategy such as internal ROS/pH-sensitive chemical bond and external ultrasound trigger could be meticulously considered in future. To realize optimal therapeutic effects, choosing appropriate biomimetic DDS according to the demands is of significance.

For now, the clinical translation of biomimetic DDS for IS treatment is still tempered by hurdles such as safety, complexity and unsatisfactory therapeutic efficiency. Therefore, more efforts should be made to further investigate the detailed properties biomimetic nanoparticles.

## Data Availability

Not applicable.

## Abbreviations

IS: Ischemic stroke
BBB: Blood brain barrier
DDS: Drug delivery system
LUV: Large unilamellar vesicles
EAATs: Excitatory amino acid transporters
GPCR: G-protein coupled receptor
ROS: Reactive oxygen species
RNS: Reactive nitrogen species
mPTP: Mitochondrial permeability transition pore
MMP: Matrix metalloproteinase
ECM: Extracellular matrix
CCBs: Calcium channel blockers
GABA: Gamma amino butyric acid
DAMPs: Danger-associated molecular patterns
PRR: Pattern recognition receptor
GFAP: Glial fibrillary acid protein
BDNF: Brain-derived neurotrophic factor
GDNF: Glial cell line-derived neurotrophic factor
NGF: Nerve growth factor
VEGF: Vascular endothelial growth factor
CNTF: Ciliary neurotrophic factor
NT-3: Neurotrophin 3
EPO: Erythropoietin
TREM2: Triggering receptor expressed on myeloid cells 2
PLTs: Platelets
SIRPα: Signal regulatory protein alpha
NSCs: Neural stem cells
MSCs: Mesenchymal stem cells
EV: Extracellular vesicles
